# Choosing a suitable method for the identification of replication origins in microbial genomes

**DOI:** 10.3389/fmicb.2015.01049

**Published:** 2015-09-30

**Authors:** Chengcheng Song, Shaocun Zhang, He Huang

**Affiliations:** ^1^Department of Biochemical Engineering, School of Chemical Engineering and Technology, Tianjin UniversityTianjin, China; ^2^Key Laboratory of Systems Bioengineering, Ministry of Education, Tianjin UniversityTianjin, China; ^3^Collaborative Innovation Center of Chemical Science and EngineeringTianjin, China

**Keywords:** replication origin, EMSA, Dnase I footprinting, SPR, RIP mapping, ITC, ChIP, ChIP-seq

## Abstract

As the replication of genomic DNA is arguably the most important task performed by a cell and given that it is controlled at the initiation stage, the events that occur at the replication origin play a central role in the cell cycle. Making sense of DNA replication origins is important for improving our capacity to study cellular processes and functions in the regulation of gene expression, genome integrity in much finer detail. Thus, clearly comprehending the positions and sequences of replication origins which are fundamental to chromosome organization and duplication is the first priority of all. In view of such important roles of replication origins, tremendous work has been aimed at identifying and testing the specificity of replication origins. A number of computational tools based on various skew types have been developed to predict replication origins. Using various *in silico* approaches such as Ori-Finder, and databases such as DoriC, researchers have predicted the locations of replication origins sites for thousands of bacterial chromosomes and archaeal genomes. Based on the predicted results, we should choose an effective method for identifying and confirming the interactions at origins of replication. Here we describe the main existing experimental methods that aimed to determine the replication origin regions and list some of the many the practical applications of these methods.

## Introduction

**FIGURE 1:**
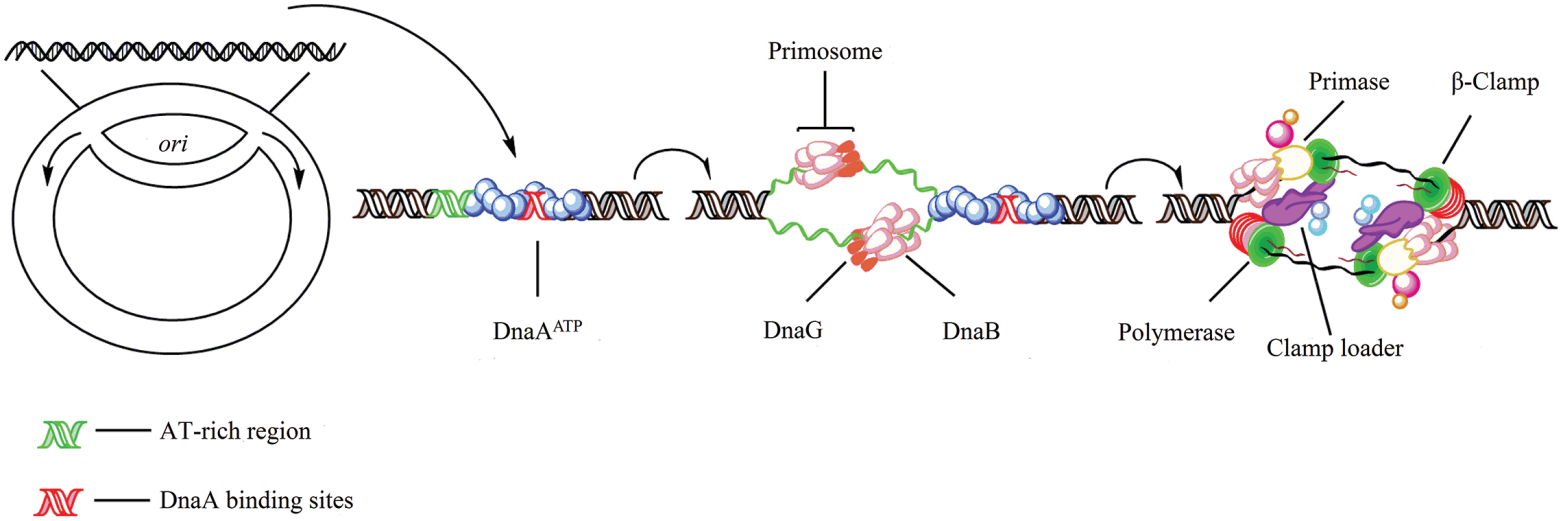
**Initiation of replication in bacteria.** In *Escherichia coli*, replication initiation requires binding of the DNA-binding protein DnaA to DnaA-boxes at the chromosome origin *oriC* which is regulated by SeqA (Dame et al., 2011). Then, with the activation of ATP, two DnaB hexamers and the helicase loader DnaC, one double hexamer for each replication direction, are positioned by DnaA into the loop (Wahle et al., 1989; Skarstad and Katayama, 2013). Primase (DnaG) which can enter the complex and synthesize two leading strand primers, stimulates release of the regulatory protein DnaC from DnaB after transiently binding to the DnaB replicative helicase (Arias-Palomo et al., 2013). Also, DnaB binds to the sliding clamp loader, a ring-shaped dimer of the β-subunit which in turn binds the DNA polymerases III (Kelman and O’Donnell, 1995; O’Donnell et al., 2013).

**FIGURE 2:**
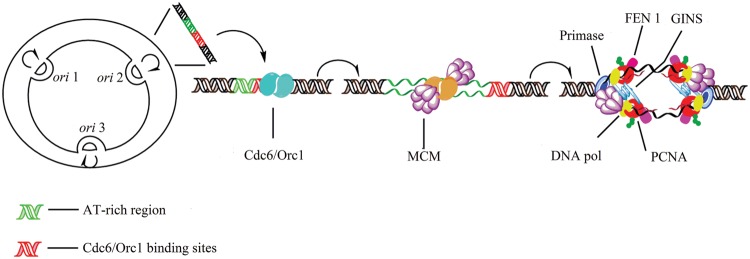
**Initiation of replication in archaea.** Archaeal circular chromosome can contain a single or multiple origins (*oriC*). Archaea have the AAA+ Orc1/Cdc6 origin-binding protein, which shares extensive sequence homology with eukaryotic ORC proteins (Zhang et al., 2009). Minichromosome maintenance (MCM) proteins bind preferentially to the *oriC* region. ATP bound Cdc6/Orc1 associates with the Cdc6/Orc1-origin complex and with the MCM helicase. Following ATP hydrolysis the Cdc6/Orc1 protein releases the helicase, and the primase replaces the Cdc6/Orc1 protein binding to MCM. MCM interacts with the archaeal GINS (*g*o, *i*chi, *n*ii, *s*an [five, one, two, three in Japanese]) complex (Marinsek et al., 2006) which is additionally capable of binding primase. Each DNA Pol interacts with a trimer of PCNA (proliferating cell nuclear antigen). The flap endonuclease FEN1 and DNA ligase I are only assembled to PCNA clamp of similar structure to *E. coli* β (Michel and Bernander, 2014).

Genome duplication is essential for cellular life. Since the determination of complete genome sequences of many species, attention has been given to the understanding of DNA replication. There are important differences among bacteria, archaea, and eukaryotes in the process of DNA replication, but they all have the same core components of replication machines: DNA polymerases, circular sliding clamps, a pentameric clamp loader, helicase, primase, and single-strand binding protein (SSB) (Waga and Stillman, 1998; Garg and Burgers, 2005; Johnson and O’Donnell, 2005; Barry and Bell, 2006). The number of replication origins varies in terms of different evolutionary lineages (Aves, 2009). In bacteria, a single DNA replication origin is sufficient enough to ensure complete and opportune replication of the entire genome precisely once in each cell cycle. In the case of *Escherichia coli*, bacteria often contain only a single replication origin in one chromosome although not all bacteria follow this paradigm (**Figure 1**). Similarly, in archaea, single replication origins have been found in *Pyrococcus* and *Archaeoglobus* (Myllykallio et al., 2000; Maisnier-Patin et al., 2002), two have been found in *Aeropyrum* (Robinson and Bell, 2007), three in *Sulfolobales*, four replication origins in the archaeon *Pyrobaculum calidifontis* (Pelve et al., 2012) and even multiple replication origins have been suggested in other genera, including *Methanocaldococcus* (Maisnier-Patin et al., 2002), *Halobacterium* (Berquist and DasSarma, 2003; Zhang and Zhang, 2003; Coker et al., 2009), and *Haloferax* (Norais et al., 2007). This illustrates how the events that occur at the DNA replication origins are predominant in the processes of DNA replication (Baker and Bell, 1998; **Figure 2**).

Initiator proteins were first proposed as the essential trans-acting factors for the initiation of DNA replication by Jacob et al. (1963). The initiator protein DnaA is the prerequisite protein in the process of prokaryotes DNA replication, and it plays an important role in forming an optimal initiation complex for DNA strand opening at the origin (Ozaki and Katayama, 2009). Among bacteria, the initiation of replication is best understood in *E. coli*. All functions of bacterial DnaA protein depend on its ability to bind specifically to an asymmetric 9-bp recognition sequence, the typical DnaA box: 5′-TTATNCACA-3′. The interactions DnaA binding to 9-mer DnaA boxes of the *oriC* is a high-affinity interaction (*K*D = 1 nM) (Speck and Messer, 2001). The sequence of *oriC* usually consists of an array of several DnaA boxes and AT-rich regions. About 10–20 DnaA molecules form a homomultimeric initiation complex on the chromosomal replication origin, *oriC*. DnaA (52 kDa) consists of four functional domains, I, II, III, IV (Messer, 2002). The ssDNA-binding activity of DnaA domain I is weak (Abe et al., 2007), but the interactions between domain I and several proteins, including domain I itself, DnaB helicase, and the initiation stimulator DiaA, are required for DnaB helicase loading onto *oriC* open complexes flexible linker (Weigel et al., 1999; Felczak and Kaguni, 2004; Abe et al., 2007; Keyamura et al., 2007; Nozaki and Ogawa, 2008). Domain III plays a major role in ATP and ADP binding, in ATP-dependent conformational changes of the DnaA multimer on *oriC*, in binding ssDNA of the *oriC* duplex unwinding element (DUE), and in ATP hydrolysis (Katayama, 2008; Ozaki et al., 2008). The C-terminal domain IV (∼10 kDa) has a typical helix–turn–helix fold that binds to DnaA box (Fujikawa et al., 2003). Domain IV Arg399 recognizes three more base pairs (5′ one-third of the DnaA box sequence: TTA) by base-specific hydrogen bonds in the minor groove of DNA (Fujikawa et al., 2003). Mostly, the C-terminal DnaA (IV) that was fused to a tag such as His_6_ or GST in the C-terminus or N-terminus is necessary and sufficient for specific DNA binding (Richter and Messer, 1995; Roth and Messer, 1995; Sutton and Kaguni, 1997; Blaesing et al., 2000). DnaA binds to high- or low-affinity sites of origin and forms an oligomeric structure (Kawakami and Katayama, 2010) that involves two types of DnaA–DNA interactions, a double-stranded and a single-stranded DNA (Speck and Messer, 2001; Ozaki and Katayama, 2012). Furthermore, the DnaA protein is not only an initiator that binds to the specific site *oriC* but it is also a gene regulatory protein. There are about 300 high-affinity DnaA binding sites and a very large number of low-affinity sites around the chromosome (Kitagawa et al., 1996; Roth and Messer, 1998). Also, replication of microbial chromosome(s) occurs via the concerted action of many other origin binding proteins (oriBPs) which are cooperative with bacterial DnaA. The oriBPs includes factor for inversion stimulation (Fis), integration host factor (IHF), sequestration factor A (SeqA), aerobic respiration control (ArcA), inhibitor of chromosomal initiation (*lciA*) and that which binds specifically site(s) to *oriC* (Wolański et al., 2014). As reports have shown, only tens of origin regions of eubacteria and archaea have been confirmed experimentally (Myllykallio et al., 2000; Maisnier-Patin et al., 2002; Berquist and DasSarma, 2003; Matsunaga et al., 2003; Lundgren et al., 2004; Robinson et al., 2004; Norais et al., 2007; Coker et al., 2009).

A number of computational tools based on various skew types have been developed for predicting replication origins. Chromosome replication origins were mapped *in vivo* in the two hyperthermophilic archaea of *Sulfolobus acidocaldarius* (Duggin et al., 2008) and *Sulfolobus solfataricus* (Lundgren et al., 2004; Robinson et al., 2004), as well as in *Haloarcula hispanica* (Wu et al., 2013), *haloarchaeon Halobacterium* sp. NRC-1 model (Coker et al., 2009), *Pyrobaculum calidifontis* (Pelve et al., 2012), *Nitrosopumilus maritimus* (Pelve et al., 2013), and *Haloferax mediterranei* (Pelve et al., 2013), using high-throughput sequencing-based marker frequency (MF) analysis. MF analysis has been successfully used in combination with microarrays to study replication characteristics and to map chromosome replication origins in both bacteria (Khodursky et al., 2000) and eukaryotes (Raghuraman et al., 2001). Recently, the Web-based system Ori-Finder^1^ and Ori-Finder 2^2^ which utilize the Z-curve method and comparative genomics analysis were used to find *oriC*s in bacterial and archaeal genomes, respectively with high accuracy (Zhang and Zhang, 2005; Gao and Zhang, 2008; Gao, 2014; Luo et al., 2014). Ori-Finder 2 is also able to analyze the unannotated genome sequences by integrating them with gene prediction pipelines and BLAST software for gene identification and function annotation. The predicted *oriC* regions from Ori-Finder have been organized into an online database DoriC^3^, which contains *oriC*s for >2000 bacterial genomes and 100 archaeal genomes, respectively (Gao and Zhang, 2007; Gao et al., 2012, 2013). Based on the predicted results, we can identify and confirm the *oriC* by its interaction with the initiator protein DnaA, and by its ability to form higher-order structures with DnaA that can be seen in the electron microscope.

Over the past several years, the rapid development of techniques used for confirming protein–DNA interaction *in vivo* and *in vitro*, such as gel retardation assay, surface plasmon resonance (SPR), electrophoretic mobility shift assays (EMSA), the DNase I footprinting technique, replication initiation point (RIP) mapping, isothermal titration calorimetry (ITC), chromatin immunoprecipitation (ChIP), and ChIP sequencing (ChIP-seq) have resulted in an increasingly refined picture of the biochemical rules governing protein–DNA interactions. Protein-DNA interactions can be explored by various *in vitro* and *in vivo* strategies, which present different advantages and disadvantages. This review begins with a discussion of the main existing experimental methods that are applied to verify protein–DNA interactions *in vivo* and *in vitro*, as well as explore some functional components of the complexes, especially applied in detecting transcription factor binding sites. Then, we outline the main advantages and limitations of these methods in **Table 1**. Through the listed methods, we could choose the most suitable experimental strategy for identifying replication origins.

**Table 1:** Summary of main experimental methods.

Methods	Benefits	Drawbacks	Extends
Electrophoretic mobility shift assay (EMSA)	(1) Qualitatively and quantitatively. (2) Effectively when protein at low concentration. (3) High sensitivity (capable to resolve complexes of different protein or DNA by stoichiometry, even to detect conformational changes). (4) Particularly useful for analyzing protein–DNA interactions on a small fragment (20–30 bp) and for determining cell types that contain each binding activity.	(1) Protein–DNA interaction must survive in gelelectrophoresis. (2) Disrupting specific interactions due to large amount of non-specific competitor. (3) Intact complexes to be highly stabled.	(1) MC-EMSA; (2) Supershift EMSA (SS-EMSA). (3) The two-color electrophoretic mobility shift assay. (4) The microfluidic mobility shift assays (MMSAs). (5) Reverse EMSA and the antibody supershift assay. (6) EMSA followed by SDS-PAGE with Western blot detection or followed by 2DE and MS were developed to identify the uncertain binding proteins. (7) Several classes of quantitative affinity-based microfluidic EMSAs including immunoassays (IAs), affinity EMSAs, and dragtag-based EMSAs. (8) EMSA linked with nanoparticle–aptamer conjugates (NP-EMSA)
Dnase I footprinting	(1) Quantitative. (2) Effective primarily when protein is at high concentration. (3) Identify DNA-binding proteins in crude extracts qualitatively. (4) Resolving the microscopic binding affinities containing multiple-binding sites with which DNA-binding proteins interact cooperatively.	(1) Binding must be stable only in solution. (2) Disrupting specific interactions by MgCl_2_ and CaCl_2_ which are required for DNase I activity.	(1) A method by using 96-well plates and capillary electrophoresis for high-throughput analysis of protein-binding sites in DNA. (2) GeF-seq (genome footprinting by high-throughput sequencing) combines *in vivo* DNase I digestion of genomic DNA with ChIP coupled with high-throughput sequencing.
Surface plasmon resonance (SPR)	(1) Label-free; (2) Versatility; (3) High sensitivity; (4) No purified sample. (5) No background interference. (6) Biological sample without tags. (7) Real-time monitoring dynamic response process. (8) Particularly useful for scanning a large DNA fragment (50–200 bp). (9) Flexibility (SPR sensors can perform continuous monitoring as well as one-time analyses)	(1) High cost. (2) Limitations in specificity of detection (biomolecular recognition elements may exhibit cross-sensitivity to structurally similar but non-target molecules) and sensitivity to interfering effects (includes adsorption of non-target molecules by the sensor surface and sample temperature and composition fluctuations).	(1) SPR-CELLIA system. (2) An automated system to analyze protein complexes by integrating a SPR biosensor with highly sensitive nanoflow liquid chromatography–tandem mass spectrometry (LC–MS/MS). (3) Localized-SPR (LSPR.) (4) A label-free method to immobilize basic proteins onto the C1 chip for SPR assay at physiological pH. (5) SPR effect of CD-Ag nanoparticles. (6) Spectroscopic SPR and imaging SPR.
Replication initiation point (RIP) mapping	(1) Faster technique and less laborious. (2) More reliable and sensible to compare between a wild-type protein and its mutants. (3) Observing and measuring the association and dissociation of the complex, and Gibbs free energy difference. (4) 1000-fold more sensitive and effective to separate the nascent DNA and nicked contaminating DNA by selective degradation of 5′ DNA by λ-exonuclease prior to primer extension.	(1) Only mapping the initiation point of eukaryotic and some archaea which have short eukaryotic or eukaryotic-like Okazaki fragments	
Isothermal titration calorimetry (ITC)	(1) Label-free; (2) Highly sensitive; (3) In solution; (4) The only method capable of determining the enthalpy, entropy, and the Gibbs free energy of a reaction in a single titration experiment.	(1) Modeling is limited to systems obeying simple Michaelis–Menton kinetics.	(1) In conjunction with complementary techniques such as X-ray crystallography, NMR spectroscopy, small angle X-ray scattering (SAXS), circular dichroism spectroscopy (CD), intrinsic fluorescence and immunoisolations; (2) the Omega ITC, MCS ITC, VP-ITC, Auto-ITC, Nano ITC-III, and ITC200 instruments
Chromatin immunoprecipitation (ChIP)	(1) Real-time monitoring dynamic response process; (2) The only way which allows one to determine the entire spectrum of DNA binding sites for any given protein *in vivo* with whole-genome DNA microarrays.	(1). Difficultly to find the specific antibody that the assay needs. (2) Necessarily to demonstrate the expression of target protein under the intracellular simulation environment.	(1) ChIP-Seq; (2) Re-ChIP; (3) ChIP-chip which combines ChIP and microarray technology. (4) ChIPon tiled arrays (ChIPOTle). (5) Insertional chromatin immunoprecipitation (iChIP). (6) Engineered DNA-binding molecule-mediated chromatin immunoprecipitation (enChIP).


## Conventional Methods for Detecting Protein–DNA Interaction at Origins of Replication *In Vitro*

### Electrophoretic Mobility Shift Assay

**FIGURE 3:**
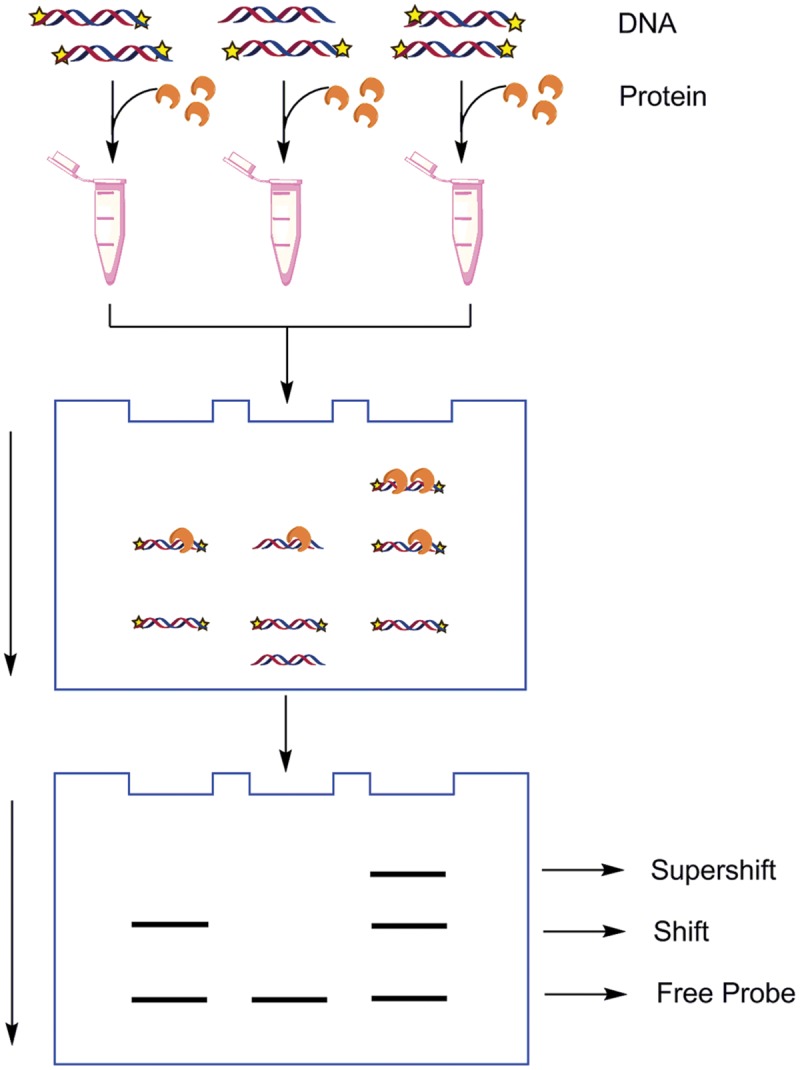
**The schematic illustration of electrophoretic mobility shift assays (EMSA).** Typically one compound is labeled to follow its mobility during electrophoresis. In general, a single protein binds to a single site. Once the length of the nucleotides is sufficient for the binding of two or more proteins, the protein–DNA complexes migrate as distinct bands, usually referred to as a super shift. If the labeled nucleotides are bound by the proteins, then the mobility of the labeled nucleotides through the electrophoretic medium will be retarded.

The EMSA, also known as the band shift, gel shift, or gel retardation assay (Lane et al., 1992), is one of the most sensitive and straightforward methods to determine the binding site-size of the DNA binding protein using a series of DNA polymers even when the protein is at a low concentration within the extract (Carey et al., 2012). It is based on the principle that DNA/RNA–protein complexes migrate more slowly when subjected to non-denaturing polyacrylamide or agarose gel electrophoresis as compared to unbound free probe (**Figure 3**). The DNA probes used may be radiolabeled or dyes specific to stain DNA and protein may be used to visualize the DNA/RNA–protein interaction. In general poly (dI-dC) is added to abolish any non-specific binding. Polyacrylamide gels offer better electrophoretic resolution for protein–DNA and protein–RNA complexes of Mr ≤ 500,000 than agarose gel (Fried, 1989). Experimental procedures, announcements and guides for troubleshooting the most common problems that we have encountered were described detailedly by Hellman and Fried (2007) and Carey et al. (2013b).

The preponderances of EMSA account in large part for the application of a wide range of conditions and the continuing popularity of the assay. This assay can be applied to a wide range in size and structure of nucleic acids and proteins binding. Lengths from short oligonucleotides to several 1000 nt/bp of single-stranded, duplex, triplex, and quadruplex nucleic acids as well as small circular DNAs, and proteins size from small oligopeptides to transcription complexes with *M*_r_ ≥ 10^6^, all of these conditions are applicable in EMSA (Hellman and Fried, 2007; Alves and Cunha, 2012). EMSA also works well with both highly purified proteins and uncharacterized binding activities present in crude protein extracts (Memelink, 2013). Low concentrations (0.1 nM or less) and small sample volumes (20 μL or less) (Hellman and Fried, 2007) are performed by EMSA due to using radioisotopes to label nucleic acids and autoradiography. Variants or the assay using fluorescence, chemiluminescence, and immunohistochemical detection are also available though less sensitive than radioisotopes.

Since its first publication in 1981, several improvements and variant techniques of EMSA have been developed. Reverse EMSA (rEMSA) and the antibody supershift assay were applied for identifying DNA–protein interactions (Tsai et al., 2012). EMSA followed by SDS-polyacrylamide gel electrophoresis with Western blot detection (Granger-Schnarr et al., 1988; Chen and Chang, 2001) or followed by two-dimensional electrophoresis (2DE) and mass spectrometry (MS) (Woo et al., 2002; Stead et al., 2006) were improved to identify the uncertain binding proteins. The supershift EMSA (SS-EMSA) can identify proteins that carrying a specific epitope in mobility-shifted complex(es) and validate previously identified proteins. Supershift EMSAs suggested the presence of transformation-specific DNA replication complexes in transformed human cells (Di Paola et al., 2010). MC-EMSA is a competition-based method developed by Smith and Humphries (2009) to identify unknown DNA binding proteins incubated with a pool of unlabeled DNA consensus competitors prior to adding the labeled DNA probe. A sensitive two-color EMSA was developed by Jing et al. (2003) for detecting both nucleic acids and protein that either free or bound conditions in gels. This assay is fast, simple, and needless the use of radioisotopes. The microfluidic mobility shift assays (MMSAs) as quantitative EMSA utilize affinity molecular probes (target) to induce a change in analyte molecule size and/or charge (Fourtounis et al., 2011; Karns et al., 2013). Several classes of quantitative affinity-based microfluidic EMSAs including immunoassays (IAs), affinity EMSAs, dragtag-based EMSAs, and other were elaborated by Pan et al. (2014). A separation technique for DNA–protein complex which called microchip electrophoretic mobility shift assay (μEMSA), based on EMSA by microchip electrophoresis was developed by Inoue et al. (2011). The performance of EMSA linked with nanoparticle–aptamer conjugates (NP-EMSA) was improved over the traditional EMSA (Wang and Reed, 2012). The most striking advantages of NP-EMSA as described in this research are real-time detection of protein–oligonucleotide interactions, the avoidance of harmful radioisotopes, and elimination of the need for expensive gel imagers.

Electrophoretic mobility shift assays is by far the most frequently used for detecting *oriC*-DnaA or -oriBPs complexes, ARS–ORC complexes, largely because it is technically the easiest and is often the most sensitive. The proteins which required in EMSA could be obtained from either purified proteins or crude extracts of cells. And the length of target DNA used in EMSA is best less than 300 bp. So, the electrophoresis separation effect of probe and protein–DNA complexes will be more obvious. Particularly, EMSA is useful for analyzing protein-DNA interactions on a small fragment (20–30 bp). So, EMSA could be used for identifying the interactions between oriBPs and *oriC*s, as well as the interactions between oriBPs and single or multiple DnaA boxes (Schaper et al., 2000; Zawilak et al., 2003; Robinson et al., 2004; Pei et al., 2007). For instance, by EMSA, the DnaA of *Thermoanaerobacter tengcongensis* was detected that it could achieve the efficient binding at a lower protein concentration (8 nM) when the DNA fragment containing two DnaA boxes with 3-bp spacing at 60°C, and the domain IV of DnaA is thermo-adaptive (Pei et al., 2007). All most the published papers for identifying origins of replication applied EMSA as the basic strategy as well as a standard to determine whether to do the following experiments.

### DNase I Footprinting

**FIGURE 4:**
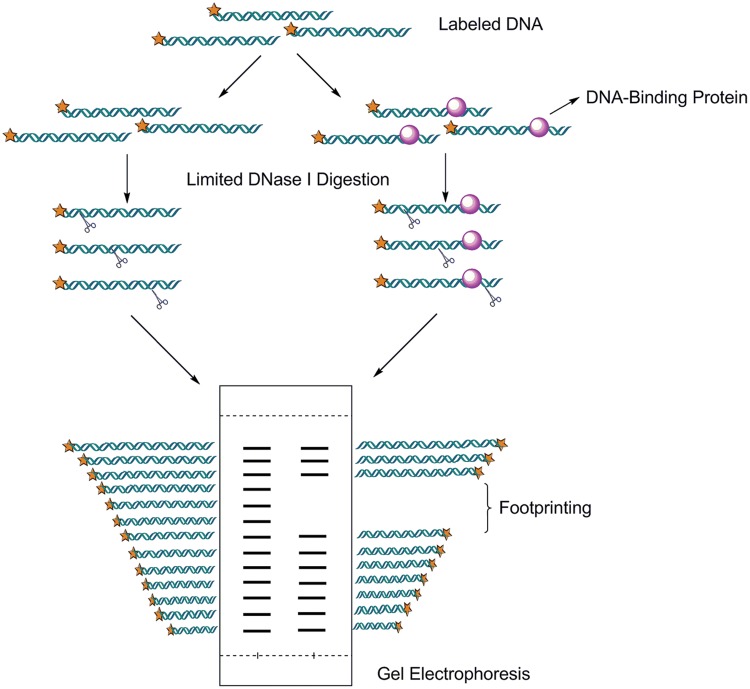
**Dnase I footprinting.** This Analysis involves endonuclease treatment of an end labeled DNA fragment bound to a protein. This technique relies on the fact that fragments of DNA that have DNA-binding proteins bound will move more slowly through an acrlyamide gel. The enzyme DNaseI will only cut exposed DNA. Limited digestion yields fragments terminating everywhere except in the footprint region, which is protected from digestion.

The second most common assay is DNase I footprinting, although its use is rapidly declining. The limitation of this method is that it doesn’t provide identity of the protein and requires higher concentration protein than EMSA (Leblanc and Moss, 2001). Even so, this method provides a myriad of applications both in determining the site of interaction of most sequence-specific DNA binding proteins and characterizing the binding interactions. The protein–DNA complexes are separated from free (unbound) DNA relies on a change that the protein prevents binding of DNase I in and around its binding site and thus generates a “footprint” in the cleavage ladder in denaturing acrylamide gel (shown in **Figure 4**). The distance from the end label to the edges of the footprint represents the position of the protein-binding site on the DNA fragment. In addition of DNase I, the enzymatic digestion methods also include the use of MNase (Fox and Waring, 1987), methidiumpropyl-EDTA_Fe(II) (MPE) (Van Dyke and Dervan, 1983), copper phenanthroline, uranyl photocleavage, hydroxyl radicals, DMS, and iron complexes (Dey et al., 2012). The classic experimental procedure, recipes, and consideration were detailed by Carey et al. (2013a). Recently, DNase I footprinting assay with fluorescent 6-carboxyfluorescein (FAM)-labeled probes was widely used for identifying the correct nucleotides regions that proteins protected (Zianni et al., 2006). The use of FAM-labeled primers eliminates the need for radioactively labeled nucleotides, slab gel electrophoresis, as well as commonly available automated fluorescent capillary electrophoresis instruments. The result of Thermo Sequenase outputted by Genemapper software was accurately aligned with DNase I digestion products, providing a ready means to assign correct nucleotides to each peak from the DNA footprint. Genome Footprinting by high-throughput sequencing (GeF-seq) was proved powerful to elucidate the molecular mechanism of target protein binding to its cognate DNA sequences (Chumsakul et al., 2013). In this research, GeF-seq combines *in vivo* DNase I digestion of genomic DNA with ChIP coupled with high-throughput sequencing.

Different with EMSA, DNase I footprinting is useful for scanning a large DNA fragment (50–200 bp) for DNA–protein interaction. Mostly, DNase I footprint assay was used for initially identifying the location and number of DnaA boxes from the whole region of *oriC* after EMSA. Through high-throughput analysis, the sequences of DnaA boxes could be confirmed and analyzed. DNase I footprinting widely applied in identification of *oriC*s in bacteria and archaea. The two *oriC*s of *S. solfataricus* have been identified before, DNase I footprinting assay has been fully used in the study (Robinson et al., 2004). Through DNase I footprinting, the precise sequences and locations of ORBs (origin recognition boxes) in *oriC*1 and *oriC*2 of *S. Solfataricus* which bind to three Orc1/Cdc6s have been directly identified, respectively. DNase I footprinting was also used for the identification *oriC*s of *E. coli* (Fuller et al., 1984), *Pyrococcus furiosus* (Robinson et al., 2004), and *Caulobacter crescentus* (Taylor et al., 2011). So, DNase I footprinting is one of the most useful method for identifying replication origins in microbial genomes.

### Surface Plasmon Resonance

**FIGURE 5:**
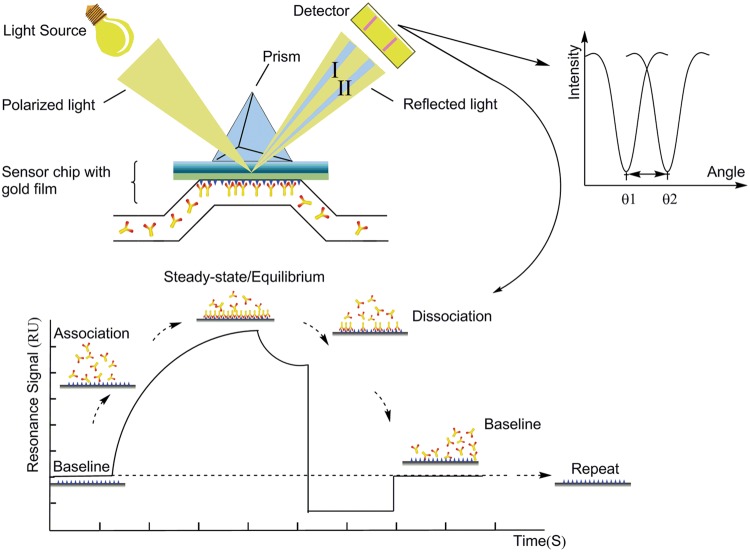
**The schematic illustration of surface plasmon resonance (SPR) system.** SPR detects changes in the refractive index in the immediate vicinity of the surface layer of a sensor chip. The sensor surface is gold with antibodies attached to it. During the measurement, the chip is irradiated from the bottom with a beam of a wide angle range within that of total internal reflection. The SPR angle shifts (from I to II in the diagram) when biomolecules binding events cause changes in the refractive index at the surface layer. The detector will determine the angle of the intensity decrease. This change in resonant angle can be monitored non-invasively in real time as a plot of resonance signal (proportional to mass change) versus time (Sawhney and Singh, 2000; Cooper, 2002; Pattnaik, 2005; Sahai, 2011; Hou and Cronin, 2013).

Since the SPR (surface plasmon resonance) technology was first used in chemical sensors, SPR sensors have gradually become an emerging alternative to the conventional *in vitro* techniques to study DNA–protein interactions, due to its label-free, high-sensitivity, real-time analysis, and flexible system design (Liedberg et al., 1995; Homola et al., 1999; Ladd et al., 2009). **Figure 5** depicts the basic principle and schematic illustration of SPR system. Compared to other methods studying protein interaction, such as direct protein interaction *in vitro* and co-immunoprecipitation, SPR is a more sensitive and quantitative biophysical approach that can measure binding affinity and kinetics simultaneously (Hoa et al., 2007). Furthermore, this technique is the basis of many lab-on-a-chip and biosensor applications. According to recent research, SPR technology can be particularly used to study the interactions between nucleic acids or protein-nucleic acids by real-time tracking of the nucleic acid reaction process. This application of SPR is unmatched by other techniques (Pattnaik, 2005; Sahai, 2011). The stoichiometry and kinetics of complex formation between DnaA protein and *oriC* could be analyzed using SPR experiments.

Surface plasmon resonance technique is an optical method for measuring the refractive index of very thin layers of material adsorbed on a metal. Its development will further extend the potential of SPR-sensing technology and allow SPR sensors to be used far more widely. Spectroscopic SPR and imaging SPR have been further adapted as affinity detection techniques in the proteomic and genomic fields, especially in a protein conformation study (Despeyroux et al., 2000), biomarker profiling, aptamer selections (Murphy et al., 2003), and antibody selections (Wilson and Howell, 2002). SPR-CELLIA system was configured for either whole cells or macromolecules in two parallel flow paths (Baird and Myszka, 2001). Applied Biosystems has also launched Affinity Sensor instrument based on SPR technology (Pattnaik, 2005). An automated system which developed for analyzing protein complexes by coupling a polymerization initiator to a biospecific interaction and inducing inline atom transfer radical polymerization (ATRP) was developed with highly sensitive nanoflow liquid chromatography-tandem mass spectrometry (LC–MS/MS) (Liu et al., 2010). Nanomaterials developed for localized surface plasmon resonance (LSPR) are increasingly integrated to classical prism-based SPR sensors, providing enhanced sensitivity and lower detection limits (Bolduc and Masson, 2011). Khan et al. developed a label-free method to immobilize basic proteins onto the C1 chip for SPR assay at physiological pH, which presents ligand with less conformational modification and thereby maintains the ligand at optimal biological activity (Khan et al., 2012). Besides, some materials have been proposed to improve the performance of SPR biosensors, such as gold nanoparticles, magnetic nanoparticles (MNP), carbon nanotubes, electropolymerized molecularly imprinted polythiophenes (Lyon et al., 1998; Wang, 2005; Parab et al., 2010; Pernites et al., 2010; Špringer et al., 2014).

Surface plasmon resonance-based biosensing is one of the most advanced label free, real time detection technologies. But, one of the main drawbacks that stem further development of SPR applications is the lack of sufficient sensitivity to reliably detect small changes in refractive index caused by compounds with low molecular weight or in low concentration at the sensing surface (Wang, 2005). So, several approaches have been reported to resolve such limitations. A modified SPR device achieved that the plasmonic detected single molecules in real time without the need for labeling or amplification by using a gold nanorod. And, the sensitivity of this device is ∼700 times higher than state-of-the-art plasmon sensors (Zijlstra et al., 2012). A new approach to SPR biosensors for rapid and highly sensitive detection of bacterial pathogens is based on the spectroscopy of grating-coupled long-range surface plasmons (LRSPs) combined with MNP assay (Wang et al., 2012). A highly efficient SPR immunosensor was effectively enhanced the sensitivity by using a non-covalently functionalized single graphene layer on a thin gold film (Singh et al., 2015).

DNA fragments were immobilized on a streptavidin matrix coated sensor chip by biotin covalent linkage. SPR analysis was performed by injecting solutions of replication origin protein from targeted bacteria or archaea followed by injection of replication origin protein from other bacteria or archaea for comparison (Jiang et al., 2003; Pei et al., 2007). Also, we can use SPR for analyzing the binding reactions of ATP- and ADP–DnaA protein to the *oriC* regions (Schaper et al., 2000; Pei et al., 2007). Based on the difference functions of ATP and ADP, the result revealed that DnaA proteins require ATP for site-specific unwinding at *T*. *tengcongensis oriC* region (Pei et al., 2007). Similar result was obtained in *Thermus thermophilus* (Schaper et al., 2000). This is similar to those in *E. coli* and *T. maritima*, further supporting that the ATP dependent activation of DnaA in replication initiation is highly conserved in bacteria. The study of *S. solfataricus* eukaryote-like Orc1/Cdc6 initiators interacting with DNA polymerase B1 (Zhang et al., 2009) and *T. tengcongensis* DnaA initiators interacting with anti-terminator NusG (Liu et al., 2008) also profited from the widespread use of SPR. Messer et al. (2001) studied DnaA rules for DnaA binding and roles of DnaA in origin unwinding and helicase loading by SPR.

### Replication Initiation Point mapping

**FIGURE 6:**
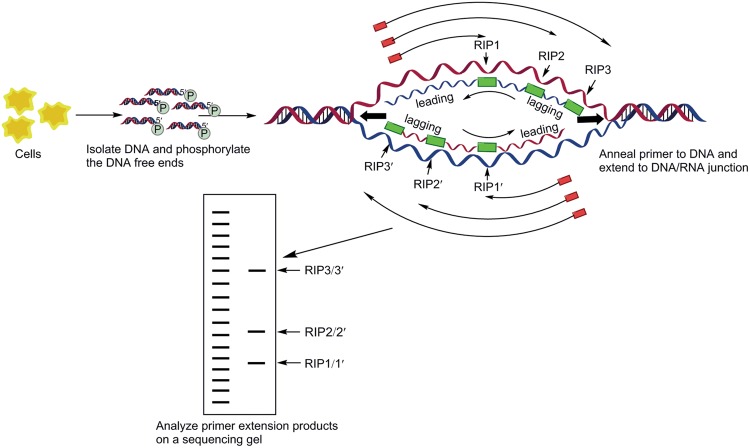
**Replication initiation point (RIP) mapping.** The replication bubble of semi-discontinuous replication is diagrammed here. After phosphorylation of any 5′-OH ends with polynucleotide kinase, replication intermediates enriched on the BND-cellulose column are treated with λ-exonuclease to digest nicked DNA. Digestion is confirmed on the agarose gel before proceeding to the primer extension reaction. The primer extension products are showed as arrows outside the replication bubble, stopping at DNA/RNA junctions on the DNA. Extension stops at the points labeled RIP1, RIP2, RIP3. Green rectangles depict the RNA primers of nascent strands. PCR products are purified and analyzed on a denaturing polyacrylamide gel. Due to asynchrony, the replication bubble can be of various sizes, resulting in various length, is the transition point from discontinuous to continunous synthesis. Sequencing and RIP reactions were analyzed side by side on the same gel (Gerbi and Bielinsky, 1997; Matsunaga et al., 2003; Lee and Romero, 2012).

Replication initiation point mapping method was developed by Gerbi and Bielinsky (1997) and Bielinsky and Gerbi (1998) to identify the RIPs by using the symmetry of a typical replication bubble that emerges once the bidirectionally moving forks have been established. This technique has been successfully used to detect the initiation sites of DNA replication (even locations of each DnaA box) at the nucleotide level in chromosomes (Matsunaga et al., 2003; Robinson et al., 2004; Pei et al., 2007) or plasmid (Sun et al., 2006) in many organisms. RIP mapping utilizes the shortest lengths of eukaryotic Okazaki fragments to map the transition point between leading and lagging strand synthesis by extending primers to various initiation points in an asynchronous population of replicating molecules schematic illustration (as shown in **Figure 6**). The extension products are fractionated on sequencing gels finally where maps that leading strand synthesis starts at a unique site, in both small and large origins.

Replication initiation point mapping is 1000-fold more sensitive and more effective to separate the nascent DNA and nicked contaminating DNA by selective degradation of 5′ DNA by λ-exonuclease prior to primer extension (Gerbi and Bielinsky, 1997) which ensures the integrity of RNA-primed DNA. Incipiently, this technology was used to identify the RIP of Eukaryote. Recently, works were demonstrated that archaea also have short eukaryotic-like Okazaki fragments allowing this technique to be used to map the initiation point of *P. abyssi* (Matsunaga et al., 2003). Robinson et al. (2004) performed RIP mapping to identify two origins of replication (*oriC*1 and the Cdc6-3 proximal origin-*oriC*2) in the single chromosome of the hyperthermophilic archaeon *S*. *solfataricus*. RIP mapping confirmed that the autonomously replicating sequence (ARS) elements corresponding to each replicon were functional in the chromosomal context of the halophilic archaeon *Haloferax volcanii* (Norais et al., 2007). But, because the exact size of the RNA primer synthesized by archaeon primase *in vivo* is not known, this technique does not allow the identification of the precise nucleotide at which replication initiates in archaeon that have multi-*oriC*s.

### Isothermal Titration Calorimetry

**FIGURE 7:**
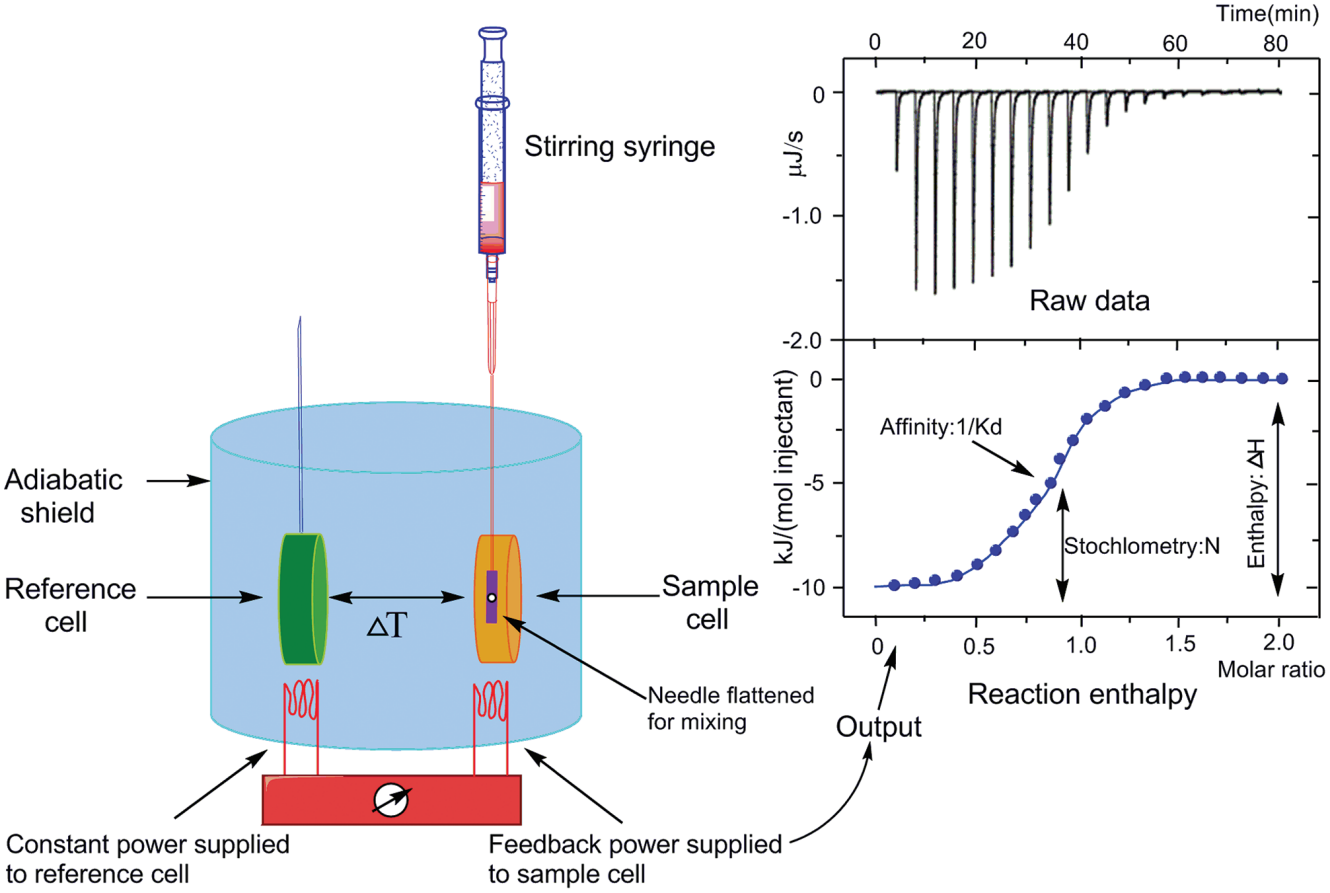
**Basic principle of isothermal titration calorimetry.** Schematic representation of the isothermal titration calorimeter (left) and a characteristic titration experiment **(upper right)** with its evaluation **(lower right)**. In **(upper right)** picture, the titration thermogram is represented as heat per unit of time released after each injection of the ligand into the protein (black), as well as the dilution of ligand into buffer (blue). In **(lower right)** picture, the dependence of released heat in each injection versus the ratio between total ligand concentration and total protein concentration is represented. Circles represent experimental data and the line corresponds to the best fitting to a model considering *n* identical and independent sites. The syringe is inserted in the sample cell and a series of injections are made (Freyer and Lewis, 2008; Martinez et al., 2013).

Isothermal titration calorimetry is a label-free, powerful, and highly sensitive technique for studying molecular interactions in solution. This method has been applied quite extensively to investigate the interaction of a macromolecule (in general, a protein) with small ligands (Sigurskjold, 2000; Velazquez-Campoy and Freire, 2006), other proteins (Pierce et al., 1999; Velazquez-Campoy et al., 2004), and nucleic acids (Matulis et al., 2000) as well as with drugs (Ward and Holdgate, 2001; Boonsongrit et al., 2008) and metal ions (Zhang et al., 2000), relies on the fact that such an interaction is accompanied by a heat effect. It does not rely on the presence of chromophores or fluorophores, nor does it require an enzymatic assay. A number of parameters such as enthalpy of binding (ΔH), entropy of binding (ΔS), association constant (Ka), binding stoichiometry (n), free energy of binding (ΔG), and potential site–site interactions (cooperativity) can be obtained from a single calorimetric titration, providing a full thermodynamic description of an interacting system (**Figure 7**).

Isothermal titration calorimetry has been one of the most common tools used for investigating interactions of protein association with nucleic acids. Recent advances in ITC instrumentation and data analysis software like the Omega ITC, MCS ITC, VP-ITC, Auto-ITC, Nano ITC-III, and ITC200 instruments have facilitated the development of experimental designs. It also can provide an informative thermodynamic when used in conjunction with complementary techniques such as X-ray crystallography, NMR spectroscopy, small angle x-ray scattering (SAXS), circular dichroism spectroscopy (CD), intrinsic fluorescence, and immunoisolations. Many particularly interesting reports employ ITC, with a focus on protein interactions with nucleic acids. Zhou et al. (2008) have utilized ITC in their study of the role of *E. coli* proline utilization A (PutA) flavoprotein, which acts as the transcriptional repressor of proline utilization genes putA and putP. ITC of PutA binding to the optimal oligonucleotide (O2) revealed a strongly endothermic interaction in Tris buffer but a weakly exothermic interaction in phosphate buffer. Kozlov and Lohman (2012) employed ITC to analyze the interaction about *E. coli* SSB and *D. radiodurans* SSB binding to ssDNA, respectively. Crane-Robinson et al. (2009) and Gilbert and Batey (2009) present an overview of ITC experiments on protein/DNA complexes, with detailed descriptions of the experimental methodologies. This review concentrates on the thermodynamics of interaction of protein DNA binding domains with DNA duplexes, and gives a thorough description of the joint implementation of ITC and differential scanning calorimetry (DSC) to provide a thorough description of the binding process. In spite of the widely using, there remain some important points to the use of ITC that should always be considered. Just as Falconer said in two reviews about ITC (Falconer et al., 2010; Falconer and Collins, 2011), several aspects of ITC data collection have been outlined in the reviews.

As more and more correlative analyses are performed and databases increased their informative capacity, ITC should develop more accurate and powerful for estimating binding affinities from known structures and conversely to use thermodynamic data to make informed predictions regarding the properties of molecular interfaces. Although ITC is widely used in identification of protein–DNA interaction, the using in identification of replication origins is vacant.

## Conventional Methods for Detecting Protein–DNA Interaction at Origins of Replication *In Vivo*

### Chromatin Immunoprecipitation

**FIGURE 8:**
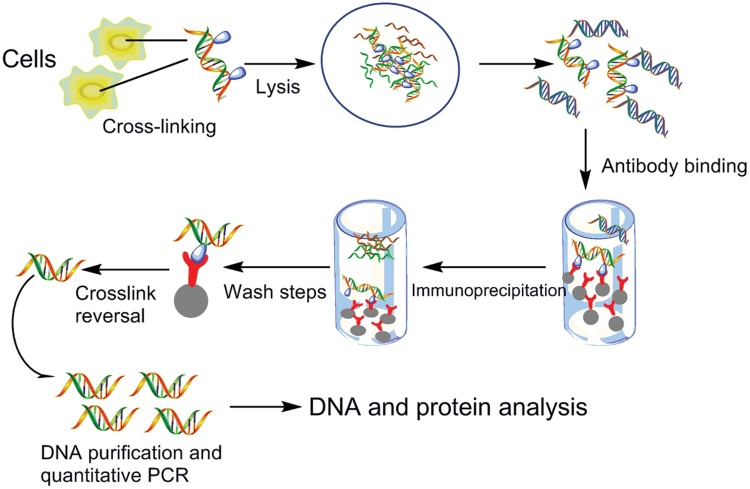
**Chromatin immunoprecipitation.** Protein and associated chromatin in living cells or tissues are temporarily bonded, the DNA–protein complexes (chromatin-protein) are then sheared into ∼500 bp DNA fragments using either enzymatic digestion or physical shearing by sonication. Cross-linked DNA fragments associated with the protein(s) of interest using formaldehyde are selectively immunoprecipitated from the cell debris using appropriate protein-specific antibody. After the cross-links are reversed, the associated DNA fragments are purified and their sequence is determined. These DNA sequences are supposed to be associated with the protein of interest *in vivo*. The DNA undergoes PCR amplification using primers targeting a particular genomic locus. These DNA sequences can be subjected to a number of downstream analysis techniques, including targeted approaches, like semiquantitative PCR and quantitative PCR, and genome-wide analyses using microarrays (ChIP–chip) and deep sequencing (ChIP-seq), ChIP-on-chip (Shah, 2009; Vinckevicius and Chakravarti, 2012).

Chromatin Immunoprecipitation is an excellent experimental method to determine the interactions of proteins with their binding sites *in vivo*. This technique is frequently used to detect the interactions between DnaA or oriBPs and replication origins due to the ability that ChIP assays allow one to determine the entire spectrum of DNA binding sites for any given protein *in vivo* with whole-genome DNA microarrays. ChIP also could be used for determining whether there were changes in the levels of binding *oriC*s and DnaA during different cell-cycle phase *in vivo* (Robinson et al., 2004; Duggin et al., 2008). As described in many papers, living cells should be handled with chemical cross-linkers to covalently bind proteins with each other and then with their DNA targets. Once cross-linked to associated proteins, sonication is used to extract and fragment chromatin, and specific antibodies against a target protein is employed to isolate protein–DNA complexes. The cross-links that is binding with proteins and DNA are then reversed, and the associated DNA was subjected to qPCR analysis to test for coprecipitation of specific DNA sequences (Orlando, 2000; Buck and Lieb, 2004). Using specific antibody or several antibodies together is one of key steps in ChIP assay. Antisera was obtained through recognizing one major chromatin associated band of approximately expected molecular weight in cell-free extracts of *Pyrococcus furiosus* (Komori and Ishino, 2001), *S*. *solfataricus* (Robinson et al., 2004), *C. crescentus* (Gorbatyuk and Marczynski, 2005; Taylor et al., 2011), and *Pyrococcus abyssi* (Matsunaga et al., 2001, 2007). The anti-DnaA antibody of *E. coli* (Sekimizu et al., 1988; Newman and Crooke, 2000) and *Bacillus subtilis* (Ogura et al., 2001; Gorbatyuk and Marczynski, 2005) was obtained by the same way. In a study of the identification of the the chromosomal *dif* site that binds Xer in *S. solfataricus in vivo* via ChIP and ChIP–chip, the antibodies required in ChIP assay were affinity purified from antisera that were raised against Xer-6H (His6-tagged Xer) in rabbits using Xer-6H immobilized on an NHS-activated agarose Hi-Trap column (GE Healthcare) (Duggin et al., 2011). The basic method is shown in **Figure 8**.

Despite the tremendous value of ChIP methods, it is important to be aware of their limitations. Carey et al. (2009) has listed three limitations of ‘standard’ ChIP experiment: (1) The ChIP assay often yields low signals in comparison to negative controls, which can lead to inconclusive results; (2) it is difficult to determine the precise binding site for a factor because of the limited resolution of the assay; and (3) ChIP is not a functional assay and cannot by itself demonstrate the functional significance of a protein or modified histone found to be located at a genomic region of interest. Recent advances in ChIP methodology have overcome some of the limitations, and the development of complementary assays, and analyses have expanded the number, types and resolution of protein–DNA interactions that have been discovered. Such as ChIP–chip (Horak and Snyder, 2002; Buck and Lieb, 2004), ChIP on tiled arrays (ChIPOTle) (Buck et al., 2005), ChIP-Seq (Robertson et al., 2007; Schmidt et al., 2009), ChIP-PaM (Wu et al., 2010), Re-ChIP (Truax and Greer, 2012) were developed for analyzing the more specific interactions between protein and DNA sequences. By means of ChIP coupled with hybridization on a whole genome microarray (ChIP–chip), researchers detected the binding of Cdc6/Orc1 to *oriC* of archaeon *P*. *abyssi in vivo*. And it was the first time that ChIP–chip method used for identifying the genome-wide distribution of the initiator of DNA replication in Archaea and Bacteria (Matsunaga et al., 2007). ChIP-on-chip was widely applied to genome-wide analysis, which combines the specificity of ChIP with the unbiased, high-throughput capabilities of microarrays (Testa et al., 2005; Huebert et al., 2006; Wyrick et al., 2009; Kim et al., 2014). Isolation of specific genomic regions retaining molecular interactions is necessary for their biochemical analysis. Insertional ChIP (iChIP) was a useful tool for dissecting chromatin structure of genomic region of interest. This technique can efficiently isolate of specific genomic domains (Hoshino and Fujii, 2009). In addition, a novel method called engineered DNA-binding molecule-mediated chromatin immunoprecipitation (enChIP) was established, for purification of specific genomic regions retaining molecular interactions (Fujita et al., 2013). Here, we detailed analyze ChIP-seq.

### ChIP Sequencing

Chromatin immunoprecipitation coupled with microarrays (ChIP–chip) or short-tag sequencing (ChIP-seq) has become the standard technique for identifying the locations and biochemical modifications of bound proteins genome-wide. ChIP-seq can be done without prior knowledge of the underlying sequence and relies only on the subsequent DNA sequence alignment to the reference genome of interest Compared to ChIP–chip. Furthermore, the nature of the microarray hybridization signal makes detection and rigorous quantification of low abundance signals problematic. Taken together, ChIP-seq can provide greater resolution, sensitivity, and specificity compared to ChIP–chip (Johnson et al., 2007; Robertson et al., 2007; Schmidt et al., 2009). Owing to the tremendous progress in next-generation sequencing technology including the Genome Analyzer (Illumina, formerly Solexa), SOLiD (Applied Biosystems), 454-FLX (Roche), and HeliScope (Helicos) (Morozova and Marra, 2008; Schmidt et al., 2009), ChIP-seq offers higher resolution, less noise, and greater coverage than its array-based predecessor ChIP–chip. With the decreasing cost of sequencing, ChIP-seq has become an indispensable tool for studying gene regulation and epigenetic mechanisms.

ChIP-seq experiments generate large quantities of data, and effective computational analysis will be crucial for uncovering biological mechanisms. An important consideration in experimental design is the minimum number of sequenced reads required to obtain statistically significant results. The standards and guidelines for carrying out ChIP-seq has been described based on the collective experience of laboratories involved in the Encyclopedia of DNA Elements (ENCODE) and model organism ENCODE (modENCODE) projects, including antibody validation, choosing appropriate sequencing depth, experimental replication, data quality assessment, data and metadata reporting (Landt et al., 2012). However, ChIP-seq has been proved to be a potential tool in the study of histone modifications, nucleosome positioning, and mapping of binding sites of various DNA binding proteins. Certainly, there are more and more researchers used ChIP coupled with high-throughput sequencing (ChIP-seq) to identify replication origins precisely, especially for the yeast genome or other eukaryotes (Eaton et al., 2010, 2011; Gilbert, 2010; Martin et al., 2011). Using ChIP or ChIP-seq, we can capture the change of DnaA protein level in the whole replication process of cells *in vivo.*

### Other Methods and Applications

In addition to the methods described here, many methods were developed to identify the majority of origins found in the previous report. Complements and extends were achieved by direct, high resolution mapping of potential origins and proteins that could bind with the specific sites in the origins of replication, also something related to replication origins.

Owing to the pivotal role played by DNA-associating proteins in various cellular processes, many *in vitro, in vivo, in silico*, and biophysical techniques have been developed to study DNA–protein interactions. *In vitro* technique includes southwestern assay, yeast one-hybrid assay (Y1H), phage display and proximity ligation assay (PLA); scanning probe microscope (SPM) is a novel *in vivo* method on the interaction of protein–DNA; biophysical technique includes many methods, such as fluorescence-based techniques [time-resolved fluorescence depolarization, double labeled native gel electrophoresis and fluorescence-based imaging, fluorescence resonance energy transfer (FRET) techniques (Clegg, 1995)], capillary electrophoresis with laser-induced fluorescence (CE-LIF) (Riddick and Brumley, 2008), also some fluorescence-based protein or nucleic acids bioprobe like FRep (Shahravan et al., 2011), quantum dots (QDs) (Michalet et al., 2005), SPR, nuclear magnetic resonance, circular dichroism (CD), atomic force microscopy (AFM), and microcalorimetry (Dey et al., 2012).

ARS (autonomously replicating sequence) assays first utilized to prove that DNA sequences was important for replication by determining whether a given DNA fragment initiates replication when placed on a plasmid in *yeast* (Struhl et al., 1979). The plasmid-based ARS assay was used to identify numerous replication origins in budding and *fission yeasts* (Newlon, 1996; Huberman, 1999). PCR-based assay which is an alternative approach to the plasmid-based ARS assay was utilized to identify replicator at ectopic sites in the genome (Malott and Leffak, 1999; Vernis et al., 1999; Tao et al., 2000). In 1996, EMSA and DNase I footprint analysis were employed to detect the interaction of the IciA protein which is known to bind to the AT-rich repeat region in the *E. coli* origin of chromosome replication, with AT-rich regions in replication origins of plasmids F and R1 (Wei and Bernander, 1996). The direction of replication fork movement is ascertained to pinpoint the origin located between the outwardly moving forks by neutral/alkaline gel electrophoresis (Nawotka and Huberman, 1988). Patrizia Contursi first described the functional cloning of a chromosomal *oriC* of the hyperthermophilic archaeon *S*. *solfataricus* from an archaeon and confirmed the proposed location by 2-D gel electrophoresis experiments. As described in the study, it represented an important step toward the reconstitution of an archaeal *in vitro* DNA replication system (Contursi et al., 2004). 2D neutral–neutral agarose gel analysis was used to test whether the loci associated with the cdc6 genes in the single chromosome of *S. solfataricus* might contain origins of replication (Robinson et al., 2004). Due to DNA isolated from asynchronously replicating cells and subjected the DNA to digestion with restriction enzymes, this technique can detect replication intermediates directly corresponding to the resolution of distinct arcs on the gel. Furthermore, RIP mapping was used to identify the RIPs at both origins in *S. solfataricus* and DNase I footprinting analysis, ChIP, EMSA were all utilized frequently to detect whether the Cdc6 could bind to the both origins in this study (Robinson et al., 2004). Zawilak et al. (2003) have presented the DNA recognition properties of the *H. pylori* DnaA protein. The interactions between the purified DnaA protein of *H. pylori* and its target were analyzed by gel retardation assay and SPR *in vitro*. A series of competition gel retardation assays were performed to elucidate the binding requirements and analyze the DNA–protein complexes (Zawilak et al., 2003). In the study of mechanism for the DnaA-*oriC* cooperative interaction at high temperature and duplex opening at an unusual AT-rich region in *T*. *tengcongensis*, many techniques for studying the interaction of protein–DNA complexes were utilized for different purposes. The GAL4-based yeast two-hybrid system, EMSA, RIP mapping, open-complex formation assay, SPR, nuclease P1 assay were used in this research for different interactions of protein–DNA complexes. It’s proud that it’s the first experimental demonstration of the chromosomal RIP in thermophilic bacteria at nucleotide level (Pei et al., 2007).

In the study of interactions of DnaA proteins from distantly related bacteria with the replication origin of the broad host range plasmid RK2, DNase I footprinting, gel mobility shift, and SPR analyses were utilized to compare the interactions of *oriV* with five different DnaA proteins from *E. coli, Pseudomonas putida, Pseudomonas aeruginosa, B. subtilis*, and *Streptomyces lividans* (Caspi et al., 2000). The results revealed that the DnaA proteins of a host bacterium were incapable to form a stable and functional complex with the DnaA boxes at *oriV* is a limiting step for plasmid host range (Caspi et al., 2000). Mode of initiator-*oriC* interactions with the loop formation between the subcomplexes of the discontinuous origin of *H. pylori* was revealed by the experimental analysis of RIP mapping, electron microscopy, and immunoprecipitation assay. *H. pylori oriC* exhibited bipartite structure and being the first such origin discovered in a Gram-negative bacterium (Donczew et al., 2012). Katarzyna et al. (2014) used SPR and EMSA methods to measure the sequence-specific interactions of Rep proteins with ssDNA within the DNA unwinding element (DUE) in the AT-rich region of the plasmid replication origin.

## Conclusion

The relevant information of *oriC* could be found from the *oriC* predicting tool such as Ori-Finder as well as the online databases DoriC which include the locations of replication origins sites for thousands of bacterial chromosomes and archaeal genomes. Based on the predicted results, we can identify and confirm the interactions at origins of replication by experimental methods. Of course, purifying replication relevant protein is another pivotal step for the research. An ideal method would require minimal cell numbers or purified protein, could be able to detect rare interactions with high specificity and sensitivity, as well as it could be easily modified to quantify interactions and provide complete information on either of protein or DNA. *In vitro* techniques provide better quantitative characterization but require isolation of active, soluble protein, which can be challenging and impractical in high-throughput assays. Additionally, protein function may depend strongly on assay conditions; hence, a non-native *in vitro* environment can give rise to results contradictory to those performed in an *in vivo* assay. Alternatively, *in vivo* assays provide a native-like environment for studying the protein–DNA interaction. Due to the restriction of experimental conditions both *in vivo* and *in vitro*, as showed in the review, more than one method were applied in most of experiments to measure the multiple protein–DNA interactions that take place in and around replication origins. And outstanding results were received by them.

However, the sequence of replication origins must be known in methods of EMSA, SPR, ITC, and DNase I footprinting. ChIP and ChIP-seq detect replication origin interactions genome-wide under the condition of unknown or known binding sequences. Through ChIP-seq, the binding sequences could be confirmed precisely. And, the most important point is that we can visually observe the amount change of oriBPS during the cell cycle. Thus the results could help us to understand the mechanisms and regulations of microbial replication initiation clearly. As was showed in the research about how DnaA and essential response regulator CtrA compete to control *C. crescentus* chromosome replication, previous EMSA experiments was used for single DnaA binding site targets (G1 DnaA box), then DNase I footprinting assay was applied to identify replication origin (*Cori*) sites (G1, G2, W1, W2, W3, W4, W5) protected by DnaA and the position of CtrA binding site ‘e’ (Taylor et al., 2011). From the figure of autoradiograph of the sequencing gel, CtrA obscures some DnaA protected sites, and all others DnaA is displaced by CtrA binding. The result of DNase I footprinting assay showed the weaker binding ability of DnaA proteins of *C. crescentus* than CtrA. The followed ChIP assay *in vivo* and western blot showed that DnaA is continuously present during the cell cycle, and CtrA proteolysis coincides with DnaA binding to *Cori*. Therefore, series of assays proved that DnaA is regarded primarily as a chromosome replication regulator and secondarily as a transcription regulator, CtrA is regarded primarily as a transcription regulator.

These methods have promoted the development in this field, however, a numerous of problems need to be solved timely. Many techniques were explored to detect the interaction of protein and nucleic acids, while how to improve these techniques to employ in the study of replication origins will be the further work that we do. Hence, we envisage that progress in these technologies will further improve detection abilities and allow sensitive, fast, and cost-effective biochemical analysis both in laboratories and in the field. This development will further extend the potential applications and allow them to be used far more widely. With the development of science and technology and strong cooperation between the various disciplines, research strategy with innovative thinking and novel methods will continue to emerge. It can be predicted that research on the regulation and mechanism of replication origins will make considerable progress in the near further.

## Conflict of Interest Statement

The authors declare that the research was conducted in the absence of any commercial or financial relationships that could be construed as a potential conflict of interest.

## Footnotes

1 http://tubic.tju.edu.cn/Ori-Finder/

2 http://tubic.tju.edu.cn/Ori-Finder2/

3 http://tubic.tju.edu.cn/doric/
